# Relationship between university students’ emotional expression on tweets and subjective well-being: Considering the effects of their self-presentation and online communication skills

**DOI:** 10.1186/s12889-023-15485-2

**Published:** 2023-03-30

**Authors:** Shaoyu Ye, Kevin K. W. Ho, Kei Wakabayashi, Yuuki Kato

**Affiliations:** 1 Faculty of Library, Information and Media Science, University of Tsukuba, Ibaraki, 305-8850 Japan; 2 Graduate School of Business Sciences, University of Tsukuba, Tokyo, 112-0012 Japan; 3 Faculty of Arts and Sciences, Sagami Women’s University, Kanagawa, 252-0383 Japan

**Keywords:** COVID-19, Emotional expression, Subjective well-being, Twitter

## Abstract

This study investigated how personal characteristics such as generalized trust, self-consciousness and friendship, and desire for self-presentation are related to the subjective well-being of university students who use Twitter in Japan, including the effects of their online communication skills. We conducted a survey in May 2021 with Twitter users and analyzed their log data between January 2019 and June 2021. The log data of 501 Twitter users, including the number of public tweets, retweets, and emotional expressions among different patterns of social media (e.g., Twitter only, Twitter + Instagram, Twitter + LINE + Instagram, etc.) and academic standings, were analyzed using ANOVA and stepwise regression analyses. The results showed that the number of tweets and retweets, with and without photos/videos, increased in 2020 and 2021 compared to 2019, and the ratio of positive sentences remained almost the same for the two-and-a-half-year period of this study. However, the proportion of negative sentences increased slightly. It is clear that the factors which affected the university students’ subjective well-being differed depending on the respective patterns of social media use.

## Introduction

Since January 2020 and the beginning of the COVID-19 pandemic, many people have experienced changes in their lives. Since then, many cities and countries have experienced lockdowns, and people were asked to physically distance themselves from each other during the quarantine and lockdown arrangements. One of the most common ways to connect with family members and friends was through the Internet, mainly via social media, to make phone calls, send text messages, and share posts with others. However, connecting with friends and relatives through social media could not fully compensate for the loss of face-to-face interaction. Previous research has indicated that people’s well-being has been affected significantly, and how they connect has changed [1].

To understand the impact of the pandemic, we examined the changes in people’s emotions through their behavior on social media. Previous research has investigated how emotional expression and posting motivation on Twitter relate to a person’s social network structure. Specifically, Kitamura et al*.* [2] examined 1,472 Twitter users aged 20–39 years, showing a significant negative relationship between social reward motivation and the number of negative emotion words, particularly anxiety emotion words. Additionally, a significant positive relationship between recording motivation and the number of positive emotion words was observed, and positive emotion words increased as the clustering coefficient increased. When the clustering coefficient exceeds the average value and increases, the coefficient of exchange/self-sufficiency to predict positive words also increases. In particular, when the clustering coefficient is low, the amount of change in exchange/self-sufficiency motivation is small but increases when the clustering coefficient is high. These findings suggest that emotional expression in Twitter posts is related to the user’s motivation and might depend on the size of the social network. The relationship between Twitter usage and personality traits is also confirmed by Mori and Haruno [3]. They built machine learning models that predicted the user’s personality using Twitter usage features and showed that word statistics information on Twitter is a good estimator of mental health traits. Their report provides strong evidence for the link between Twitter posts and personal characteristics. Additionally, Ye et al*.* [4] examined the relationship between university students’ generalized trust, social skills, number of tweets, types of emotional expressions and topics, and subjective well-being. The results indicated that: (i) users with higher levels of generalized trust and social skills had a higher level of subjective well-being and used fewer negative expressions; (ii) users with a large number of tweets used both positive and negative expressions but they used more negative than positive expression; and (iii) users who used fewer negative expressions and those who used more positive expressions had higher levels of subjective well-being. However, they only showed correlations among these factors, whether there are causal relationships or not still remains unknown. In addition, the implication from Ye et al*.* [3] is before COVID-19, it is necessary to investigate the impact of the pandemic on young generations by examining the influence of the structure of their social networks on Twitter, such as the number of followers, number of accounts followed, and emotional expression on their posts to explore further these relationships with subjective well-being and other related factors, since Twitter is the most popular opened social networking service (SNS) among young generations in Japan [5]. We believe investigating these relationships is crucial to help public health authorities worldwide understand how to develop policies to improve young generations’ subjective well-being during and after the pandemic. In particular, we focused on the following research question:

RQ: Does a change in emotional expression in tweets and/or retweets published on Twitter reflect the changes in users’ subjective well-being before and during the COVID-19 pandemic?

### Literature review and background of research

During the COVID pandemic, social media became the most convenient tool for communicating with others and the primary source of information and misinformation when lockdowns were in place [6]. Gao et al*.* [7], using data collected at the beginning of the pandemic, showed that social media exposure was positively associated with anxiety and the combination of depression and anxiety. Other research also reports that people only like to express their emotions and that there are norms of online expression of emotion based on the social media selected [8]. Some researchers have also used text mining to study how emotional valence is related to COVID-19 misinformation on Twitter and noted that misinformation was more related to negative valence [9].

Among the most common social media platforms, Twitter has been extensively investigated as a tool to probe into people’s emotions [10]. Mori and Haruno [3] examined the relationship between the information found on Twitter and the personal characteristics of people who replied to the tweets through machine learning. The results of the study showed that social network information on Twitter could accurately estimate a user’s personality. Additionally, linguistic statistical information and linguistic information about the words used can be used to estimate a person’s mental health status. In other words, it is possible to estimate a user’s characteristics from the number of tweets and the linguistic expression used for the tweets. In addition, as mentioned earlier, Ye et al*.* [4] observed the relationships between subjective well-being and emotional expression on Twitter.

Based on the above findings, it could be assumed that the contents of Twitter posts and emotional expressions might be related to individual characteristics. However, most of these findings were obtained before the outbreak of the COVID-19 pandemic. After the start of the pandemic and the declaration of a state of emergency in Japan, researchers began to observe the relationship between the anxiety of young people about COVID-19 and their tweets. For example, the Japanese media reported that negative expressions used on Twitter increased dramatically [11]. Ye and Ho [1] conducted a survey right after the lifting of the first state of emergency in Japan and reported that young people spent more time on Twitter to gain some emotional support because they tried to avoid face-to-face contact during the first state of emergency. However, compared to 2020, there were fewer limitations in 2021 and 2022, so they also observed changes in the relationship between social media use and subjective well-being from 2021 to 2022. In particular, their subjective well-being was not strongly related to their social media use [12]. Therefore, in this study, we analyzed the number of tweets and retweets and the presence or absence of changes in emotional expression before and during the COVID-19 pandemic and investigated their relationship.

Compared to face-to-face communication, the online world is characterized by visual anonymity, a lack of nonverbal information, and reduced concern about others’ perceptions of the users themselves, which encourages self-presentation [13]. Ye [5] conducted a survey with 1,681 university students who used social media, including LINE, Twitter, Instagram, and Facebook, to explore differences in social media usage among different platforms. The study showed that university students who used Twitter only showed the highest level of self-appeal and topic avoidance scores and the lowest scores for online communication skills, received the least social support from others and had the lowest level of subjective well-being. Because using social media may promote the diversification of young people’s self-consciousness and friendship [14], the effect is considered more prominent in the case of social media with high visual anonymity, such as Twitter.

To investigate the effects of the COVID-19 pandemic on young people’s online communication behaviors and subjective well-being, we used Twitter as the major tool to probe into this issue. Using sentiment analysis, we examined how different combinations of social media can affect subjective well-being. Previous studies also indicate that self-consciousness, and friendship and self-presentation [15] [16] are significant factors that influence young people’s subjective well-being; therefore, we also included these two factors. Furthermore, as people with a higher level of generalized trust tend to use Twitter to connect with strangers [1], we also examined the effects of generalized trust.

### Research method

#### Research design

From May 10 to 22, 2021, we conducted an online survey with university students in the Kanto region of Japan. A total of 1,694 students submitted their responses, and 1,681 were analyzed, as 13 were incomplete. However, only 577 participants had public tweets and/or retweets between January 2019 and June 2021. Therefore, in this study, we conducted the analysis of the data obtained from the 577 participants.

In the survey, their personal characteristics, including generalized trust toward others (α = 0.80), self-consciousness and friendship, desire for self-presentation and admiration, usage of various social media, online communication skills (α = 0.80), and subjective well-being (α = 0.86), were measured. Ye [5] conducted a factor analysis about self-consciousness and friendship and desire for self-presentation and admiration and found six subscales for self-consciousness and friendship (i.e., “self-indeterminate factor (α = 0.74)”, “self-establishment factor (α = 0.75)”, “self-independency factor (α = 0.71)”, “self-variable factor (α = 0.69)”, “dependency factor (α = 0.42)”, and “self-concealment factor (α = 0.61)”, respectively) and four subscales for the desire for self-presentation and admiration (i.e., “rejection avoidance factor (α = 0.84)”, “praise acquisition factor (α = 0.84)”, “self-appeal factor (α = 0.83)”, and “topic avoidance factor (α = 0.72)”, respectively). However, because the internal reliabilities for the “dependency factor” and “self-concealment factor” were lower than 0.65, we conducted the following analysis without these two factors. Additionally, we obtained participants’ consent to collect their log data from Twitter for further data analysis.

#### Emotional expression and topic analysis

We collected and analyzed the log data using the Twitter API. The number of tweets, number of retweets, number of tweets with photos/videos, and number of retweets of tweets with photos/videos were calculated as features. Terms including “コロナ” ( “corona” in katakana, the Japanese syllabic writing for terms in foreign languages), “corona,” and “covid” (including capitalized and non-capitalized letters)—hereinafter “COVID-19”—were set as keywords directly related to COVID-19, and the number of tweets and retweets including these keywords were calculated and analyzed.

Sentiment analysis was performed using a neural network model implemented using the flairNLP library [17]. The neural network uses a word-embedding layer and a bidirectional long short-term memory (BiLSTM) layer to convert tweets into feature vectors, and a linear transformation and Softmax function are used to classify sentences into three categories: positive, negative, and neutral. For the dataset for learning the parameters of the neural network, we used 20,000 Japanese tweets with emotion labels given by crowdsourcing through a service from Lancers, a Japanese crowdsourcing company. In the holdout verification, which estimated the prediction accuracy using part of the dataset as test data, the accuracy rates of the positive, negative, and neutral classes were 0.70, 0.56, and 0.59, respectively. The tweets of the analysis targets were divided into newline characters and regarded as sentences, and each sentence was input to the neural network to provide an emotion label. However, retweets, tweets without nouns, and tweets containing five or fewer words were excluded from the analysis. Hashtags, user-mention tags, and URLs were deleted from the text of the tweets. The percentage of sentences classified as positive or negative was calculated for each analysis target and used as the analysis target.

### Data analysis

#### Demographics of our participants

Table 1 shows the demographics of the participants who posted or retweeted. We found that approximately one-third of the participants were first-year students. Additionally, more than 70% lived alone, similar to the findings before the pandemic[5].^1^ Similar to previous findings, more time was spent on the Internet through smartphones than computers, however, this difference was narrowing.^2^ Additionally, the top three purposes of using Twitter were related to the collection of information, that is, killing time, sharing hobbies, and browsing news; the top three posted contents were common hobbies, sharing photos and videos, replying to friends, etc., which were similar to the content posted before the pandemic. Meanwhile, 42.5% of the respondents indicated that they posted on Twitter daily, and 22.5% reported that they rarely posted. The ratios of both types of respondents were higher than those before the pandemic period^3^ [5].

**Table 1:** Demographics (*n* = 577)

Items	Demographics			
Gender	Male 51.6%	Female 47.1%	Others 1.2%	
Average age	19.6 years (*SD* 1.31)			
Academic standing	First-year: 32.2%	Second-year: 28.9%		
	Third-year: 21.7%	Fourth-year or above: 17.1%		
Accommodation	Dormitory: 19.4%	Home/Relatives’ home: 20.3%		
	Apartment 58.9%	Shared: 1.4%		
Living style	Alone: 71.8%	With family/relatives: 22.5%		
	With acquaintances/lovers: 2.4%	Room shared: 3.3%		
Internet usage time by computer per day	12 h or more: 3.3%	10–12 h: 2.6%	8–10 h: 5.7%	
	6–8 h: 13.5%	4–6 h: 25.8%	2–4 h: 27.7%	
	0–2 h: 18.4%	Not used: 2.9%		
Internet usage time by smartphones per day	12 h or more: 3.1%	10–12 h: 3.5%	8–10 h: 7.8%	
	6–8 h: 20.6%	4–6 h: 27.4%	2–4 h: 26.3%	
	0–2 h: 11.1%	Not used: 0.2%		
Information about the Twitter account	Account Time: 44.8 months (*SD* 27.23)			
	Accounts followed: 544.6			
	Number of accounts: 2.71 (*SD* 1.76)			
	Number of followers 481.2			
Daily Twitter usage time	12 h or more: 0.3%	10–12 h: 0.5%	8–10 h: 1.7%	
	6–8 h: 3.1%	4–6 h: 6.2%	2–4 h: 23.1%	0–2 h: 65.0%
Content viewed on Twitter (Note 1)	Killing time: 83.2%	Information about hobbies: 80.6%		
	News: 49.9%	Conversation with friends: 42.5%		
	Review confirmation: 22.5%	Dissipating stress: 21.7%		
	Information about COVID-19: 16.8%	Job hunting: 7.5%		
	Others: 4.5%	None: 0.5%		
Content posted on Twitter (Notes 1 and 2)	Common hobbies: 56.3%	Photos and videos: 42.1%		
	Reply to friends’ Tweets: 40.9%	Maintaining friendships: 24.6%		
	Self-deprecating: 22.7%	A fulfilling life: 27.7%		
	Report-related: 14.7%	Others: 12.0%		
	Corona-related anxiety: 4.0%	Corona-related information: 3.3%		
	Job hunting information: 2.9%	Do not post: 24.3%		
Twitter post frequency	Almost daily: 42.5%	Several times per week: 15.9%		
	One time per week: 11.3%	One time per month: 7.8%	Hardly: 22.5%	

(1) Items that allow multiple responses

(2) 24.3% of respondents did not have any public posts. Therefore, the maximum percentage of content posted was 75.7%

#### Emotional expressions in tweets and retweets

The collected data were compared separately in 2019 (before the pandemic), 2020 (1^st^ year during the pandemic), and from January to June 2021 (2^nd^ year during the pandemic). We used data corresponding to the first half of the year for analysis. We matched this to the usage pattern of students as the academic year started in the first half of the year, and university students started to develop their social networks, especially first-year students. This arrangement also allowed us to match our data collection period (i.e., mid to late May) with the tweets and retweets they posted. At this stage, we removed data from 8 participants’ data as their tweet/retweet records were insufficient for us to conduct further analysis. We considered participants to be users of a particular social media platform if they spent at least 20% of their social media time on that particular platform. Of the 15 possible combinations of usage patterns, we observed nine patterns that met the following requirements: (1) LINE only (*n* = 5); (2) Twitter only (*n* = 70); (3) Instagram only (*n* = 2); (4) LINE and Twitter (*n* = 149); (5) LINE and Instagram (*n* = 11); (6) Twitter and Instagram (*n *= 17); (7) LINE, Twitter, and Instagram (*n* = 282); (8) LINE, Instagram, and Facebook (*n* = 1); and (9) all four social media platforms (*n* = 32). For further analysis, we only included Patterns 2 (*n* = 70), 4 (*n* = 149), and 7 (*n* = 282), as they are the only three patterns that account for more than 10% of the participants. The results are presented in Table 2. Regardless of the use patterns, the ratios of positive and negative sentences within these three periods showed no significant differences in the number of tweets and retweets, including those with keywords of COVID-19, from 2020 to 2021. The number of tweets, retweets, and tweets and retweets with photos/videos in the two-and-a-half-year period showed significant differences.

**Table 2:** Changes in the number of tweets/retweets and emotional expression based on social media usage patterns

Items	January to June 2019			January to June 2020			January to June 2021			ANOVA		
	**Overall**			**Overall**			**Overall**			**Overall**		
	**2**	**4**	**7**	**2**	**4**	**7**	**2**	**4**	**7**	**2019**	**2020**	**2021**
Number of tweets	74.28			170.44			468.90			114.27 ^***^		
	75.56	105.42	57.50	177.66	221.94	141.43	1,075.59	510.07	296.55	2.19	2.36	34.03 ^***^
Number of retweets	15.74			37.82			93.56			36.36 ^***^		
	20.87	18.81	12.85	30.00	53.92	31.25	187.54	106.44	63.43	.82	1.79	9.76 ^***^
Number of tweets with photos/videos	11.62			26.79			59.30			33.50 ^***^		
	14.70	14.79	9.18	23.93	40.70	20.16	118.07	77.11	35.30	1.50	2.28	14.78 ^***^
Number of retweets with photos/videos	5.34			16.16			31.15			14.02 ^***^		
	7.54	6.47	4.19	10.20	30.91	9.85	57.27	50.26	14.57	1.01	2.85	9.89 ^***^
Number of tweets related to COVID-19	0.00			0.11			0.14			15.76 ^***^		
	0.00	0.00	0.00	0.06	0.15	0.10	0.31	0.12	0.11	−	1.08	4.58 ^**^
Number of retweets related to COVID-19	0.00			0.00			0.01			1.00		
	0.00	0.00	0.00	0.00	0.00	0.01	0.03	0.00	0.00	−	.78	2.15
Ratio of positive sentences	0.35			0.33			0.31			3.05 ^*^		
	0.28	0.27	0.41	0.25	0.27	0.37	0.23	0.26	0.35	8.74 ^***^	9.68 ^***^	15.09 ^***^
Ratio of negative sentences	0.24			0.26			0.28			3.40 ^*^		
	0.23	0.24	0.24	0.32	0.26	0.26	0.32	0.28	0.27	.04	.04	.05

(1) Overall is the average of all participants (*n* = 501). Pattern 2 refers to exclusively Twitter users (*n* = 70); Pattern 4 refers to people who use Twitter and LINE (*n* = 149); and Pattern 7 refers to people who use Twitter, LINE, and Instagram (*n* = 282)

(2) ANOVA: Overall is the F-value of the ANOVA for comparison from 2019 to 2021. The individual year value is the ANOVA for comparing the three patterns in the same year

(3) ^***^p < .001; ^**^p < .01; ^*^p < .05

**Fig. 1:**
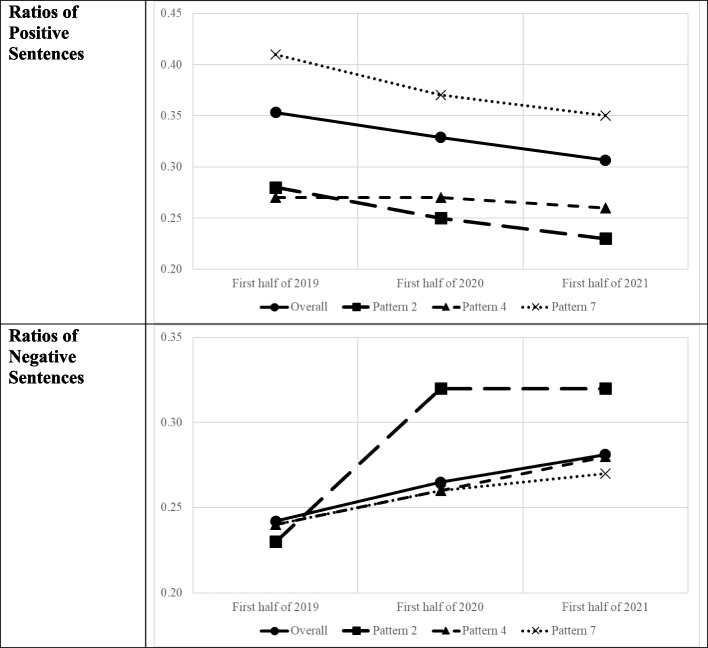
Ratios of positive and negative sentences based on social media usage patterns

As shown by Ye [5], the posting frequency of Twitter differed depending on the combination of social media platforms used, including Facebook (required users to provide a real name), Instagram (linked to Facebook), and LINE (usually used for connecting with close friends). Therefore, we analyzed the responses based on the combination of the social media platforms they use. In this study, we used Twitter, LINE, and Instagram (282 people, Pattern 7, 56.3%) as the most common patterns of social media usage, followed by Twitter and LINE (149 people, Pattern 4, 29.7%), and 70 people (Pattern 2, 14.0%) who use Twitter only. We analyzed these three patterns in detail and summarized the results in Table 2. From Table 2, it is clear that the number of tweets and retweets (F = 114.27, *p* < 0.001 for tweets and F = 36.36, *p* < 0.001 for retweets) and the number of tweets and retweets with photos/videos (F = 33.50, *p* < 0.001 for tweets and F = 14.02, *p* < 0.001 for retweets) increased from 2019 to 2021 in overall results. Therefore, there was a growing trend in the number of tweets posted and retweets (including photos/videos) from 2019 to 2021, reflecting an increase in Twitter usage. With regard to the number of tweets about COVID-19, there was a slight increase in the overall result (F = 15.76, *p* < 0.001). There were few retweets about COVID-19. Regarding the ratio of positive and negative sentences on Twitter, we noted an increase in negative sentences (F = 3.40, *p* < 0.05) and a decrease in positive sentences (F = 3.05, *p* < 0.05) in this period. Figure 1 shows the ratios of positive and negative sentences from 2019 to 2021.

**Fig. 2:**
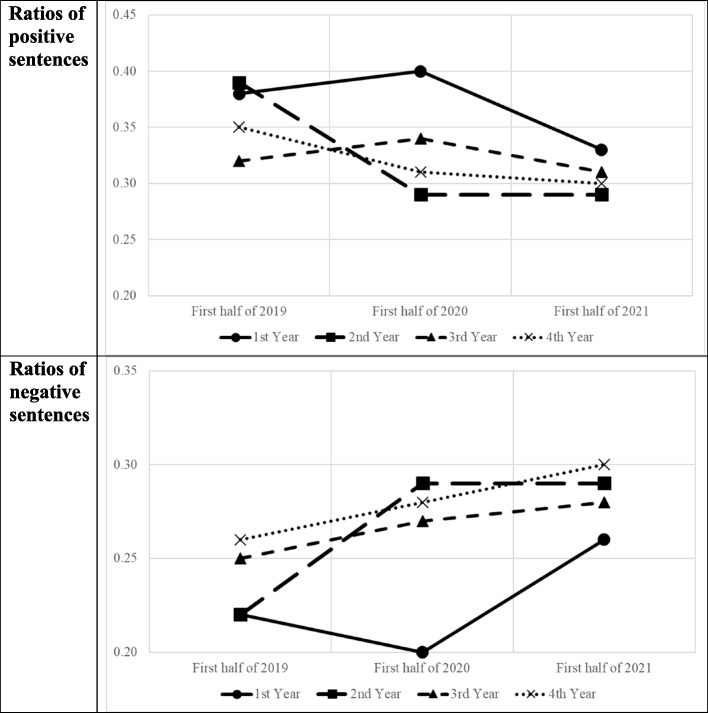
Ratios of positive and negative sentences based on academic standings

We further analyzed the data according to the academic standing of the participants (based on their academic standing in 2020–21). As shown in Table 3, we noted an increasing trend in the number of tweets, retweets, and tweets and retweets with photos/videos across academic standings. Except for fourth-year students, the average number of tweets about COVID-19 peaked in 2021. However, there was no common trend in the ratio of positive and negative sentences (Fig. 2).

**Table 3:** Changes in the number of tweets and retweets and emotional expression based on academic standing

Item	Academic Standing	January to June 2019	January to June 2020	January to June 2021
Number of tweets	First-year	50.56	72.19	422.33
	Second-year	27.48	184.89	599.26
	Third-year	98.33	238.63	405.64
	Fourth-year or higher	175.42	248.58	407.37
	F-value	8.89 ^***^	6.60 ^***^	2.13
Number of retweets	First-year	12.23	23.37	66.31
	Second-year	6.65	42.97	133.27
	Third-year	19.42	42.36	94.96
	Fourth-year or higher	34.53	51.14	73.14
	F-value	4.43 ^**^	1.18	2.75 ^*^
Number of tweets with photos/videos	First-year	8.90	17.41	46.98
	Second-year	4.91	33.14	79.58
	Third-year	16.54	27.41	55.87
	Fourth-year or higher	22.75	33.06	51.28
	F-value	5.51 ^***^	.85	1.92
Number of retweets with photos/videos	First-year	4.43	12.73	21.71
	Second-year	2.79	24.09	45.14
	Third-year	8.39	10.82	32.50
	Fourth-year or higher	7.70	15.60	22.48
	F-value	1.92	.59	1.77
Number of tweets related to COVID-19	First-year	0.00	0.01	0.10
	Second-year	0.00	0.13	0.16
	Third-year	0.00	0.17	0.17
	Fourth-year or higher	0.00	0.17	0.16
	F-value	−	3.49 ^*^	.55
Number of retweets related to COVID-19	First-year	0.00	0.00	0.00
	Second-year	0.00	0.00	0.01
	Third-year	0.00	0.01	0.00
	Fourth-year or higher	0.00	0.01	0.01
	F-value	−	1.13	.71
Ratio of positive sentences	First-year	0.38	0.40	0.33
	Second-year	0.39	0.29	0.29
	Third-year	0.32	0.34	0.31
	Fourth-year or higher	0.35	0.31	0.30
	F-value	.95	3.58 ^*^	.71
Ratio of negative sentences	First-year	0.22	0.20	0.26
	Second-year	0.22	0.29	0.29
	Third-year	0.25	0.27	0.28
	Fourth-year or higher	0.26	0.28	0.30
	F-value	.78	4.36 ^**^	.93

(1) The number of students in Year 1, 2, 3 and 4 are 162 149, 109, and 81, respectively. The F-value is the result of ANOVA for comparison between the mean value of these 4 years

(2) ^***^p < .001, ^**^p < .01, ^*^p < .05

#### Factors affecting subjective well-being

Ye [5] clarified that the self-consciousness and friendship, self-presentation desire, and online communication skills of university students differed depending on the usage pattern; therefore, we also analyzed how they differed depending on the three patterns (Table 4). There were significant differences in self-establishment (i.e., Pattern 7 [3.78] was significantly higher than Patterns 2 [3.50] and 4 [3.60], F = 3.49, *p* < 0.05), rejection avoidance factor (i.e., Patterns 2 [2.94] and 7 [2.99] were significantly higher than Pattern 4 [2.58], F = 7.59, *p* < 0.001), praise acquisition factor (i.e., Pattern 7 [3.27] was significantly higher than Pattern 4 [3.01], F = 7.32, *p* < 0.001), online communication skills (i.e., Patterns 2 [51.37] and 7 [52.83] were significant higher than Pattern 4 [48.19], F = 11.62, *p* < 0.001), Twitter usage period (i.e., Pattern 2 [52.61] was significant higher than Patterns 4 [41.49] and 7 [43.51], F = 4.21, *p* < 0.05), number of Twitter accounts (i.e., Pattern 2 [3.64] was significantly higher than Pattern 7 [2.77], which was also significantly higher than Pattern 4 [2.32], F = 13.95, *p* < 0.001), number of Twitter accounts followed (i.e., Pattern 2 [1,003.03] was significantly higher than Patterns 4 [472.11] and 7 [432.98], F = 14.36, *p* < 0.001), number of Twitter account followers (i.e., Pattern 2 [911.61] was significantly higher than Patterns 4 [338.12] and 7 [442.61], F = 13.09, *p* < 0.001), and subjective well-being (i.e., Pattern 7 [49.94] was significantly higher than Patterns 2 [43.89] and 4 [45.66], F = 12.13, *p* < 0.001) based on ANOVA and post-hoc tests.

**Table 4:** Scores for each variable for different usage patterns

Items	Overall	Pattern 2	Pattern 4	Pattern 7	F-statistics
Generalized Trust	20.23	20.26	19.85	20.43	.829
Self-indeterminate factor	3.53	3.53	3.46	3.57	.63
Self-establishment factor	3.69	3.50	3.60	3.78	3.49 ^*^
Self-independent factor	2.99	3.16	2.99	2.95	1.83
Self-variable factor	3.73	3.73	3.58	3.80	1.82
Rejection avoidance factor	2.86	2.94	2.58	2.99	7.59 ^***^
Praise acquisition factor	3.12	3.01	2.90	3.27	7.32 ^***^
Self-appeal factor	3.70	3.59	3.67	3.75	1.00
Topic avoidance factor	3.90	3.76	3.88	3.95	1.26
Online communication skills	51.24	51.37	48.19	52.83	11.62 ^***^
Twitter usage period (months)	44.18	52.61	41.49	43.51	4.21 ^*^
Twitter accounts	2.77	3.64	2.32	2.77	13.95 ^***^
Twitter accounts followed	544.53	1,003.03	472.11	438.98	14.36 ^***^
Twitter accounts of followers	477.06	911.61	338.12	442.61	13.09 ^***^
Subjective well-being	47.82	43.89	45.66	49.94	12.13 ^***^

(1) Overall is the average of three groups of participants (*n* = 501). Pattern 2 refers to exclusively Twitter users (*n* = 70); Pattern 4 refers to people who use Twitter and LINE (*n* = 149); and Pattern 7 refers to people who use Twitter, LINE, and Instagram (*n* = 282)

(2) ^***^p < .001, ^**^p < .01, ^*^p < .05

We further conducted a stepwise multiple regression using subjective well-being as the dependent variable for demographic attributes, factors related to personal characteristics, and Twitter usage (Tables 2 and 4) as independent variables. The results are presented inTable 5.^4^ As all VIF values are less than 3, our regression models did not have the multicollinearity problem. It was noted that praise acquisition positively affected the subjective well-being of students for all the three patterns. For those who used Twitter only (Pattern 2), the generalized trust had positive effects on improving their subjective well-being, whereas their self-appeal and spending more time on the Internet through smartphones lowered their subjective well-being. Similar to Pattern 2, for those who used Twitter and LINE (Pattern 4), the generalized trust had positive effects on improving subjective well-being, as well as their self-establishment. Participants of Pattern 7, those who used Twitter, Instagram, and LINE, would have a higher level of subjective well-being and self-establishment if they had a higher ratio of positive sentences and a lower level of subjective well-being if they had more Twitter accounts or had a higher level of self-indeterminism and self-independency.

**Table 5:** Factors affecting their subjective well-being between different use patterns

Factors	Pattern 2		Pattern 4		Pattern 7	
	Standardized Coefficient	VIF	Standardized Coefficient	VIF	Standardized Coefficient	VIF
Internet usage time by smartphones	−.231^*^	1.034				
Ratio of positive sentences					.159 ^**^	1.026
The number of Twitter accounts					−.113 ^*^	1.018
Generalized trust	.271 ^***^	1.192	.213 ^**^	1.055		
Self-indeterminate factor					−.150 ^**^	1.118
Self-establishment factor	.195 ^#^	1.439	.405 ^***^	1.035	.333 ^***^	1.129
Self-independency factor					− .189 ^***^	1.115
Praise acquisition factor	.385^***^	1.383	.229 ^**^	1.037	.184 ^***^	1.105
Self-appeal	−.319 ^**^	1.283				
R^2^ (adj.)	.502		.313		.308	
F-value	14.90 ^***^		23.48 ^***^		21.07 ^***^	

(1) Pattern 2 refers to exclusively Twitter users (*n* = 70); Pattern 4 refers to people who use Twitter and LINE (*n* = 149); and Pattern 7 refers to people who use Twitter, LINE, and Instagram (*n* = 282)

(2) ^***^p < .001, ^**^p < .01, ^*^p < .05

We also compared the standardized coefficients between the patterns, and the results are presented in Table 6. As noted, the results of comparisons between the coefficients were all insignificant, except for the comparison of Pattern 2 with Pattern 7 for praise acquisition.

**Table 6:** Comparison of regression coefficients

	Patterns		Unstandardized Coefficient		Degree of Freedom			SE1	SE2	SE Combined	*t*-value
	#1	#2	b_1_	b^2^	df1	df2	Combined				
Generalized trust	2	4	.707	.545	69	148	215	.242	.183	.243	.666
Self-establishment	2	4	2.498	5.011	69	148	215	1.305	.892	1.486	−1.691
	4	7	5.011	3.775	148	271	417	.892	.609	.921	1.343
	2	7	2.498	3.775	69	271	338	1.305	.609	1.325	−.964
Praise acquisition	2	4	4.806	2.606	69	148	215	1.249	.778	1.335	1.648
	4	7	2.606	2.085	148	271	417	.778	.602	.808	.644
	2	7	4.806	2.085	69	271	338	1.249	.602	1.268	2.146 ^*^

(1) Pattern 2 refers to exclusively Twitter users (*n* = 70); Pattern 4 refers to people who use Twitter and LINE (*n* = 149); and Pattern 7 refers to people who use Twitter, LINE, and Instagram (*n* = 282)

(2) ^***^p < .001; ^**^p < .01; ^*^p < .05

## Discussion

In this study, we collected personal data from university students enrolled in the Kanto region of Japan through a survey. In addition, we collected their log data (public posts) on Twitter. Then we analyzed the relationships between their social media use patterns, emotional expressions on Twitter, and subjective well-being using these variables.

### Theoretical implications

We analyzed whether emotional expression in tweets and retweets posted by university students on Twitter changed since the beginning of the COVID-19 pandemic. Additionally, we analyzed the results using social media patterns and found that the number of tweets and retweets (including tweets and retweets with photos and videos) increased from 2019 to 2021 for all major patterns. This result is probably related to the fact that about one-third of the participants were freshmen who used Twitter to build new interpersonal relationships in April 2021 when they started their university lives [5]. In 2019 and 2020, they were only second- and third-year high school students in the Japanese education system,^5^ and refrained from taking university entrance exams and seldom met their classmates during the pandemic.

In general, the effects of generalized trust, self-consciousness and friendship, and self-presentation on subjective well-being echoed previous findings. These results included: (i) a high level of generalized trust (except for Pattern 7), self-establishment (except for Pattern 2), and praise acquisition from others improved their levels of subjective well-being, and (ii) a high level of self-appeal (for Pattern 2), self-indetermination, and self-independency (the latter two for Pattern 7) reduced their levels of subjective well-being.

Regarding emotional expressions, we first analyzed the proportion of positive and negative sentences on Twitter. We noted that the proportion of positive sentences remained almost unchanged for 2.5 years, whereas the proportion of negative sentences increased slightly (Tables 2 and 3) during the same period. However, there were almost no tweets or retweets about COVID-19. In other words, many of the emotional expressions in the posts of university students are indeed negative, but it is difficult to determine whether it is due to COVID-19. Japan was one of the few nations worldwide that did not lock down the country during the pandemic; thus, its negative impact would probably be less than that of other countries. However, when further examining the proportion of positive and negative sentences based on the usage patterns and academic standings of the participants, it can be noted that there is a general trend of a decrease in the ratio of positive sentences for the overall group and based on the patterns. This may be due to the COVID-19 situation making people have bad emotions in 2020; thus, they felt unhappy.

Looking at the findings through the lens of academic standings, we noted that while first-year students had a higher ratio of positive sentences most of the time, the percentage of positive sentences saw a decrease in 2021. All three other groups also either had a decrease or flatted out from 2020 to 2021. The findings of the first-year students relate to their academic journey, as these participants were in high school in 2019 and 2020, and entered university in 2021. The reduction in the ratio of positive sentences could be due to the change in their living environment (as they would relocate from their hometown to the university and faced changes in their lives) and their attitude toward COVID-19. Indeed, our ANOVA results showed that the differences in the ratios in 2021 among the four groups were statistically insignificant.

However, there was an interesting observation regarding the ratio of negative sentences. While there was an increase in the ratio of negative sentences in 2020 for second-, third- and fourth-year students, there was a decreasing trend for first-year students. For first-year students (who were high school students in 2019 and 2020), their lives were probably not significantly adversely affected by COVID-19 in 2020. They might even have fresh experience in participating in the coursework through various types of online courses. Therefore, these new exposures might make them feel adventurous and have fewer complaints. However, second-year students might have initially experienced a lot of pressure in 2019 (as they were facing university admission examinations when the news about COVID-19 broke out in December 2019) and had a sharp increase in the ratio of negative sentences. These students probably had a more difficult time during their campus life compared to other grades, as all classes in the Spring semester were conducted online, and most classes were still conducted online in the Autumn semester. All extracurricular activities were suspended during that period, which meant that they had few opportunities to communicate with their classmates in person to reduce their anxiety and stress. As a result, we still observed an increase in the ratio of negative sentences in 2021.

We also noted that for Twitter-only users (Pattern 2), subjective well-being decreased if they spent too much time on the Internet via their smartphones. An effect of the ratio of positive sentences was observed for users who used Twitter, LINE, and Instagram (Pattern 7). The ratio of positive sentences on Twitter was positively related to subjective well-being, which may indicate that users with higher levels of subjective well-being are more likely to use more positive sentences. As Ye [5] clarified that compared to other patterns’ users, Twitter-only users received the least social support and had the lowest level of subjective well-being, while they had the highest level of depression tendency. This might be due to the highest visual anonymity on Twitter, which allows users to connect with strangers and have posts without letting other people know who they are. On the other hand, users of Twitter, LINE, and Instagram might be able to make posts freely on Twitter and communicate with strangers while communicating with their family and intimate friends through LINE, and also communicate with those friends/acquaintances who are not that intimate on Instagram. This kind of usage allows them to keep a good balance between intimate people and strangers, which helps them receive various kinds of social support (instrumental and emotional), therefore, has effects on improving their subjective well-being.

To conclude, our findings show that emotional expressions that affect subjective well-being differed depending on the pattern of use of other social media, even if all participants used Twitter.

### Practical implications

The finding of this study shows the relationships between the subjective well-being of university students in Japan and their social media use patterns. Therefore, we suggest that public health authorities should consider taking our findings in developing programs help young adults who face stress and other mental health issues due to the COVID-19 crisis [18] [19], such as using young adults’ social media use patterns as a proxy to analyze their behavior and the corresponding mental health concerns for developing better support for them.

## Conclusion

This paper presents valuable information on how university students’ emotional expressions on Twitter were related to their subjective well-being in Japan. Through the results of regression analyses, we found that subjective well-being was affected by the time spent on the Internet through smartphones (for Pattern 2), the percentage of positive sentences (for Pattern 7), and the number of Twitter accounts (also for Pattern 7).

### Limitations and future research directions

This study has some limitations. Even though 1,681 social media users participated in the survey, only 577 of them had public tweets and retweets. Additionally, among the final 501 responses, we could only analyze three of the 15 possible combinations of social media usage patterns. Furthermore, the number of tweets and retweets we collected from the respondents’ public records was insufficient to analyze their behavior further quarterly. To better understand how social media usage patterns relate to users’ subjective well-being, it would be necessary to recruit more respondents of different ages with more public tweets/retweets to analyze in more detail.

## Data Availability

The datasets used and analyzed during the current study are available from the corresponding author upon reasonable request.

## Footnotes

1 We coded “1” for living alone and “2” for living with others for the living style.

2 Monthly conversion was performed for the usage time of the Internet by computers and smartphones as follows: 1–2 h per day is 30, 2–4 h per day is 90, 4–6 h per day is 150, 6–8 h per day is 210, 8–10 h per day is 270, 10–12 h per day is 330, and 12 h or more per day is 360.

3 The frequency of post was converted monthly as follows. Rarely is 0, once a month is 0.5 times, once a week is 10, several times a week is 20, and almost every day is 30.

4 Since gender is a nominal scale, males are set to “0”, females are set to “1”, and others are set to “2” as dummy variables. Since the number of “others” was small, only men and women were included in the following multivariate analysis of this study.

5 Japanese high schools use a 3-year system.
