# Correction to: Relationship between university students’ emotional expression on tweets and subjective well-being: Considering the effects of their self-presentation and online communication skills

**DOI:** 10.1186/s12889-023-15657-0

**Published:** 2023-04-28

**Authors:** Shaoyu Ye, Kevin K. W. Ho, Kei Wakabayashi, Yuuki Kato

**Affiliations:** 1 Faculty of Library, Information and Media Science, University of Tsukuba, Ibaraki, 305-8850 Japan; 2 Graduate School of Business Sciences, University of Tsukuba, Tokyo, 112-0012 Japan; 3 Faculty of Arts and Sciences, Sagami Women’s University, Kanagawa, 252-0383 Japan

**Correction: BMC Public Health (2023) 23:594**

10.1186/s12889-023-15485-2

During the publication process the footnotes of tables 3-6 were accidentally removed. The original article has been updated to reinstate these.

